# ‘In the Midst of Every Crisis, Lies Great Opportunity’: Perceptions of the Future Use of Artificial Intelligence in the UK NHS Primary Care

**DOI:** 10.1002/msc.70092

**Published:** 2025-04-16

**Authors:** Sue Greenhalgh, Li Guo, Gill Yeowell

**Affiliations:** ^1^ Department of Health Professions Clinical Fellow Manchester Metropolitan University Manchester Metropolitan University Manchester UK; ^2^ Department of Computing and Maths Manchester Metropolitan University Manchester UK; ^3^ Department of Health Professions Manchester Metropolitan University Manchester UK

**Keywords:** AI, clinical nudge, perceptions, primary care, UK NHS

## Abstract

**Background:**

Amidst a backdrop of crisis in primary healthcare, characterised by increasing patient demands and a stagnant workforce, artificial intelligence (AI) is proposed as a potential enhancer of clinical efficacy and decision‐making support. Interviews explored how AI could serve as a ‘clinical nudge’, assisting rather than supplanting human decision‐makers.

**Method:**

This qualitative study explores stakeholder perceptions of AI in NHS primary care settings in the Northwest of England through semi‐structured interviews and site visits. Participants included healthcare professionals and patients.

**Results:**

All highlighted AI’s potential to manage large amounts of patient data that may contain inaccuracies or irrelevant information effectively, and aid in the implementation of clinical guidelines. However, concerns about data quality, cybersecurity and the impact on clinical skills were prevalent.

**Conclusions:**

Findings suggest a cautious but optimistic view of AI as a tool for improving efficiency and patient safety in primary care, emphasising the need for robust governance structures to ensure its beneficial integration into clinical workflows. This study underlines the necessity of balancing technological innovation with the preservation of essential human elements within the healthcare process.

## 1 Background

General practice is the bedrock of the National Health Service (NHS) in the UK, yet it is in crisis (Stokes‐Lampard and Openshaw 2018; Fisher 2024). It is the first point of contact for healthcare, undertaking around 4 million consultations daily. General Practitioners (GPs) and their teams delivered over 348 million appointments in 2023, 19.4 million more than the previous year (NHS England 2024). Alongside workforce shortages, demand continues to grow annually with patients presenting with increasingly complex needs (NHS Resolutions 2024). More people are living longer but in ill health, with multiple comorbidities to the end of life (Whitty 2023; ARMA 2024). A Kings Fund analysis (2016) showed that there were 30 million patient contacts from 177 practices and that consultations had grown in excess of 15% between 2010/11 and 2014/15. Over the same period, the GP workforce grew by just 4.75% (Baird et al. 2016). Furthermore, a recent study of 300,000 GP consultations in General Practice identified that the average length of a consultation was 10.9 min (Lawson 2021). Less time was spent with patients in areas of deprivation where demand and resource capacity are particularly challenged. Those with multimorbidity received an average of only 54 s longer during their consultation (Gopfert et al. 2021). General Practices are finding it increasingly difficult to recruit and retain GPs, with GPs who are reaching the end of their careers choosing to retire early in response to workload pressures. In response, First Contact Practitioner roles have been developed for healthcare professionals with advanced practice skills; for example, First Contact Practitioner Physiotherapists take on many of the musculoskeletal responsibilities carried out by GPs (Chartered Society of Physiotherapy 2018). First Contact Practitioner roles have been developing over several years; however, they too are experiencing excessive demands on their time (Greenhalgh et al. 2020).

The Darzi report (2024) offers a critical and urgent review of the NHS, highlighting a need for long‐term radical reforms including technological upgrades with an increased focus on community and preventive care. The Arthritis and Musculoskeletal Alliance (ARMA 2024) report calls for AI, data use and community‐focused healthcare to reduce the gap in health outcomes. Darzi (2024) highlights that too many people struggling with a health issue result in a hospital admission because too little time is spent in the community as a result of long‐term underinvestment in community services. A need for technology to unlock efficiency and effectiveness is called for to enhance community care. Despite the potential of AI to transform care, the use of AI in primary healthcare in the UK has challenges. This study explores stakeholder's perceptions of the use of AI in a primary care setting in the Northwest of England.

## 2 Methods

The study was reported in accordance with the consolidated criteria for reporting qualitative (COREQ) research (Tong et al. 2007). Ethical approval was obtained from the Manchester Metropolitan University Faculty Ethics Committee, UK (Ref: 68,410). Participant informed consent was obtained prior to taking part in the study.

Patient and Public Involvement and Engagement (PPIE) consultations, utilising a qualitative design from an interpretivist perspective, were undertaken to explore participants' experiences of AI in NHS Primary Care (Bourgeault et al. 2013). To gain a holistic understanding of the phenomenon of interest, semi‐structured interviews were undertaken with stakeholders with a range of experience of AI in primary care. Additionally, to understand the context of what the participants told us about AI within Primary Care, a site‐visit was undertaken to a general practitioner (GP) practice in Primary Care.

Six participants were purposively recruited through professional networks from Northwest England during August 2024. All participants were known to the team via professional contacts. Participants are selected purposefully to represent specific characteristics or experiences relevant to the research question. This approach ensured that the sample was rich in information and could provide meaningful insights (Malterud et al. 2016). It was anticipated that this sample size would be sufficient to generate rich data; however, recruitment would continue until data saturation had been achieved.

Inclusion Criteria.

- Experience of healthcare in a primary healthcare setting, and
- Experience of primary healthcare as a patient, healthcare professional, healthcare professional body representative or computer/AI expert

Exclusion Criteria.

- No access to Video Conferencing facility (Teams)

Interviews were undertaken using Teams. Interviews were undertaken by a researcher who had expertise in qualitative interviewing with experience of working in primary care (SG), alongside a second researcher with expertise in AI (LG), to facilitate the exploration of the clinical and AI perspective.

An interview schedule was used to guide the interview (see Supporting Information S1). This facilitated topics related to the aim of the study to be investigated, whilst allowing sufficient flexibility to explore new and unanticipated issues. The interview schedule was developed from a review of the literature to identify topics pertinent to the research aim. It comprised open‐ended questions supplemented with prompts to encourage detailed discussions. The guide underwent refinement through rigorous deliberation with the research team (SG, GY, LG). The questions explored during the site‐visit related to what arose from the interviews and in response to what was demonstrated by the GP practice partner stakeholder during the site‐visit. Interviews lasted between 60 and 90 min and were recorded on Teams. The site‐visit lasted 60 min and was digitally audio recorded. The audio‐recordings were transcribed verbatim by a professional transcriber to ensure accuracy of the transcription.

Data analysis was undertaken using Braun and Clarke's six phase framework for thematic analysis (Braun and Clarke 2021). This involved the team (S.G., G.Y.) independently listening to the Teams recordings and reading the transcripts to become immersed in the data. Data were then manually coded by reading the pseudonymised transcript line by line to identify salient text related to the research aim. Theoretically, cognate codes were grouped to create sub‐themes, with conceptually similar sub‐themes grouped into initial themes. The initial themes were critically reviewed and refined by the team (S.G., G.Y., L.G.) to create the final themes. Reflexive field notes of the interviewers' role and how this may have impacted the generated data were made and fed into the analysis of the findings. The team included two physiotherapists by background, both female; one worked in primary care as a consultant physiotherapist (S.G.; PhD) and the other was a researcher working in an academic institution (G.Y.; PhD). The third member of the team was a male academic working in AI (L.G.; PhD). Member checking was used to validate the findings and ensure that the findings reflected the participants' experiences.

## 3 Results

Six participants were recruited and all consented to take part in the study (Table 1). Participants included a patient and five stakeholders with a range of experience of using AI tools in health and primary care. Data saturation was achieved.

**TABLE 1:** Participant demographic information.

Demographic information
A former patient with lived experience of spinal infection who received treatment from a primary and secondary care.
A health informatics lead from the chartered society of physiotherapy (professional body for physiotherapists), who is a physiotherapist by background.
An electronic patient records expert who has developed an AI tool for physiotherapy, who is a physiotherapist by background.
A first contact practitioner who works in primary care, who is a physiotherapist by background.
A GP, and a clinical lead.
A GP practice partner stakeholder who arranged a site‐visit to a primary care GP practice

## 4 Themes

**FIGURE 1:**
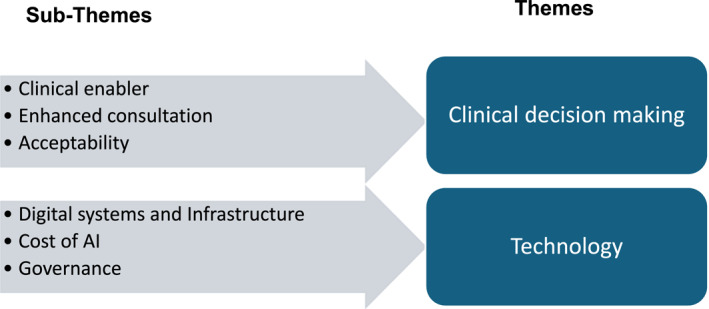
Themes and subthemes.

Two main themes were identified. The first theme ‘clinical decision making’ had three subthemes: ‘clinical enabler’; ‘enhanced consultation, ’and ‘acceptability’. The second theme ‘technology’ had the following subthemes: ‘digital systems and infrastructure’, ‘cost of AI tool’ and ‘governance’ (Figure 1). Anonymised verbatim participant quotes have been included to support each theme.

### 4.1 Theme 1: Clinical Decision Making

All participants were positive about AI and emphasised the need to introduce it into clinical practice in primary care. They felt AI could be a clinical enabler by providing a ‘clinical nudge’ to the clinician:

To the GP or the clinician, it’s there and it’s a nudge. It’s, sort of, saying, “Have you considered X, Y, Z?” If it’s going to be that type of thing [AI used in this way], then I think that’s absolutely the way forward.

P5

Additional benefits to this ‘clinical nudge’ were that it could also help as a learning tool for clinicians:

If I had a system that nudged me of all the things that I probably should have been thinking about based on the decisions that I was making, that would have been a brilliant learning tool for me as well.

P4

It was felt that this would be particularly useful for the generalist practitioner working in primary care, especially when they are faced with uncommon conditions:

We’re generalists, so being a generalist you can’t be expected to know the fine details of everything. I think having a system there that can signpost and bring things together and direct the clinician a bit, is really valuable in helping with patient safety and probably improving health outcomes.

P2

Participants emphasised that AI could support with the patient diagnosis and timeliness of this:

I think in terms of the diagnostic side it’s going to be key, isn’t it? With a lot of these pathologies that present in primary care, patients are coming in with nuances rather than severe symptoms, so it’s not like A&E where somebody might turn up with a spinal infection where they’ve got a raging infection, they look horrendous and feel really unwell. It might be the beginnings where it’s sort of quite minor symptoms that you wouldn’t necessarily automatically think are serious. It’s [AI] going to raise that awareness in that moment in time and spinal infection is obviously going to be part of that differential diagnosis.

P2

It’ll [AI] probably give better earlier diagnostics and enable clinicians to reach deeper, better‐informed decisions. If it could have been spotted earlier [my serious pathology] … prevention’s always better than cure, isn’t it? So yeah, I think it’s [AI] vitally important that that’s there.

P5

It was further observed that AI could facilitate the application of clinical guidelines in patient management by summarising these to make them more practicable for use in primary care.

You know the clinical guidance we get from NICE … They’re huge, so trying to make sense [of them], and I would challenge you to find many doctors who actually read them and use them. I just think AI could draw on all that information and actually make it useful at the practical level.

P3

Participants highlighted the potential for AI to be used early in the patient pathway to effectively triage the patients at the pre‐clinical stage to enhance timeliness:

The expectation on non‐clinical staff to do this triage, it’s a lot of pressure on the reception staff and the care navigators. And I think if you had something [AI] that gives them an additional superpower of being able to pick up on this stuff, I think it could make a big difference. Again, it has that knock‐on further down the line, doesn’t it, because you’re [the clinician] seeing people at the right time, aren’t you, and in the right place.

P2

However, whilst all participants acknowledged AI could help enhance patient safety, it was underlined that AI should only serve as a tool to assist, and not replace the clinician's decision making.

I think we still need the human being in the cockpit, there to make decisions. We can have nudges from AI to support those decisions but for the really difficult things, I think healthcare still needs a human with the hands on the steering wheel.

P4

Participants identified that one of the issues with electronic patient records was the vast amount of unstructured data.

So, when you look at past consultations, some patients are seen a few times a week. Working through their [electronic patient] records you just literally scroll, and scroll, and scroll and have to jump around into different areas [of their records] all within a 10‐ or 20‐minute appointment.

P6

The consequence of having to work through this large amount of patient data was fatiguing for the clinician:

It’s just tiring; you’re trying to optimise people’s ability to work and use the brain power in a way that's going to benefit the patient rather than doing stuff like scrolling through records. It's not good use of my brain power, trying to search through that and then feeling knackered by keep looking, because then your mind is off the serious problem in front of you.

P6

Clinical participants found that the amount of data they had to look at within a primary care consultation was sometimes overwhelming and this could make it difficult to identify pertinent information to help with timely diagnosis:

Sometimes we can get a bit overwhelmed. I certainly know new GPs find that difficult to make sense. They’re very good at getting the information, but not always very good at sorting out what's relevant.

P3

As such, they highlighted that AI could enhance the patient consultation by managing the vast amount of ‘noisy’ patient data.

Machine learning is very useful for things like noisy data, where you have a lot of data. We have a significant amount of clinical data, and a lot of it is unstructured.

P1

Others added that not only could AI help manage the data but could also identify and link important information from the patient records to facilitate an earlier diagnosis and improved patient outcomes:

So, AI could be really helpful to sift through everything and pick out some pointers for the clinician. The AI could pull all that information [from the electronic patient records] and give a summary of this information. If we’ve got the tech to help us because we’re human and there’s only so much you can do in that time. So that really is very valuable for me.

P6

Participants added that using AI could have a positive impact on patient safety:

You’re going to have the headspace, so to sort of digest the information and also what the patient is telling you. I think errors are likely to be reduced because of that increased headspace. Both at that singular appointment, but also when you look at the timeframe of the whole clinic and the whole week and whole year.

P2

The acceptability of using AI to help with clinical decision making was also raised by participants. A common concern raised regarding using AI was around the impact this technology may have on jobs:

What I hear from members [of the physiotherapy profession] is fears about “AI coming in to take our jobs and we’ll be made redundant”.

P4

It was emphasised that for AI to gain acceptance among clinicians, it should be made clear that AI is intended to assist in the decision‐making process rather than to be a replacement for the clinician.

It’s about how do you make sure that you are supporting the clinician and what they need to do and you’re not trying to take over things that they would say are key to their role. And that’s where you’ve got to try and find that balance. Because, yeah, you don’t want to step on any toes and it’s about finding a way to serve up useful information to them that helps them in their assessment, whilst also enabling them to make their own informed decisions.

P1

Moreover, it was felt that if the AI tool could show how it had arrived at its decisions, this may enhance its acceptability to clinicians:

I think people might find it difficult to accept and to trust. …AI can be a bit like a black box. You don't actually know about its thought processes. It’s sometimes important to know the processes that go on in making the decisions… having a rough idea of what it draws on and how it comes to its decisions, a bit of background. Yeah, just the clinical decision‐making process. It's just nice to have confidence, what the process has been to reach that answer.

P3

Other concerns raised were around the potential loss of clinical skills due to using AI to make clinical decisions. However, its use to ‘nudge’ the clinician rather than replacing their clinical reasoning skills was seen as an important factor in AI being acceptable to patients:

But it won’t be a skill loss because it’s not a magical AI thing that’ll do stuff. It’s just pop‐ups and nudges.

P5

It was also highlighted that professional pride and autonomy may impact its acceptability for use in clinical decision making.

Probably, pride is the main reason they wouldn’t use it. Health professionals really pride themselves on clinical judgment. … They’re very confident in their own clinical judgment and view, so they would say, “We don’t really need any help. We’re good enough anyway. Why are you saying we're not good clinicians?”

P3

Finally, trust in AI was raised as an issue that might impact the acceptability of using AI for clinical decision making.

But if you’re going to have success in healthcare then you must have a trusting relationship between the practitioner and the patient or the member of the public. So, can you build that trust as easily, using an AI device than you can in person? I don’t know.

P4

Participants also added that patients may be sceptical about the use of AI affecting the acceptability from their perspective.

Patient scepticism—they may distrust the AI. How is the use of AI going to be sold to the patients? How can patients and clinicians be sure any diagnostics are accurate? And I think there is or there can be a deep distrust of AI in general.

P5

### 4.2 Theme 2: Technology

Participants raised several potential issues around digital systems and infrastructure. Most highlighted the issue of connectivity in the NHS, both within and between primary and secondary care.

EMiS web is the standard one [platform] that’s used across [this region]. There are various other programmes that primary care use. But EMiS doesn’t talk to the hospital; it doesn’t communicate with medical records in the hospital. So, the hospital can’t see the GP records, and the GP Practice can’t see the hospital record.

P6

In addition, an ageing NHS technology infrastructure was highlighted as a potential issue to the use of AI in primary care, with participants questioning whether NHS systems could support the use of these new technologies:

So, when they’re [the clinician] needing to access some information or direct a patient to some information that might be held on a website, do they use the devices that they have in their primary care setting? I see them use what’s in your pocket than what’s on your desktop. And again, that feeds into that kind of aging infrastructure that we have in a lot of NHS, health and social care settings. Our digital infrastructure, particularly in the NHS is pretty poor. So, are we able to add on AI devices or AI into the devices that are already bursting at the seams?

P4

Others raised concerns about cybersecurity and the consequences of this in primary care:

We may place too much emphasis on AI. But as was shown with that recent outage, where they’ve been doing that security thing, all the systems went down … and I know people have got back‐up systems in place. But there seemed to be a huge issue that they couldn’t resolve quickly. And I’m just wondering how that would affect the AI [in primary care].

P5

The usability of the AI tool was highlighted as an important aspect of the usefulness of the AI tools to clinicians. It was underlined that the tool should be easy to use and that the benefits of using AI should outweigh the costs:

But it has to be easy to use, it has to be safe, they have to see the immediate value from it. …how much extra stuff does it create me from a user perspective? In terms of, I now have to log into this thing, and I have to move it over here and there’s no widget, I have to put my password in. These are things that will ultimately frustrate people, so you have to try and balance it. Does it create more value for me than it costs? And so long as you can provide the value, clinicians, they want to see that it works, that it’s not too hard to use.

P1

The cost of purchasing and integrating AI into primary care and the NHS infrastructure was raised as a potential issue by all participants:

Costs are probably going to be a barrier, potentially. There’s going to be a cost element. In terms of primary care specifically, primary care networks and GP surgeries, individual surgeries, they are businesses essentially. So, it’s whether the cost is prohibitive or not for people to use those services. Whether there’s budgets available for that.

P2

Others queried who would be liable for this cost, especially as the upfront monetary cost would fall on primary care, with the cost savings being made later in the patient pathway in secondary care:

And the challenge might be that it might not be the primary care team that see the financial benefit or see the value of delivery of it. …And yes, it’s potentially, the cost might be in primary care and the saving, or the risk mitigation might be in another part of health and social care. …So, am I paying for a benefit that somewhere else in health and social care are seeing?

P4

Several issues concerning governance were highlighted, specifically around patient safety and clinical negligence:

AI and liability; might lead into litigation—it’s a challenging area. So, putting it really bluntly, who carries the can for an AI device? So, the AI device misses a spinal infection, who carries the liability for that? Would it be the developers of the product? Would it be the trust or the organisation, the ICB, the primary care network …is it the individual clinician or are the devolving litigation because they’re deploying a tool? So, it just starts to get into a slightly grey area, and I don’t know where we stand with that.

P4

Added to this was the uncertainty around the regulation of data:

These AI devices, where they’re delivered, they may be cloud‐based but the data has to land somewhere, so where is that landing, is that UK? Are the data centre subject to the UK data regulations as opposed to those of other countries and there might be differences?

P4

Participants also highlighted that there could be potential issues with the quality of the data used by the AI tool in primary care. This deficiency in data quality could lead to health inequalities. As such, it was underscored that more needs to be done to improve the quality and quantity of data to mitigate against increasing health inequalities:

AI is a voracious consumer of data. So, it requires enormous datasets on which we can base its algorithms and we can train it and we can retrain it. So, we don’t have that great a dataset at the minute. So, all the different languages, the ethnicities, the health literacy, all those different elements of people are not represented well in the NHS dataset. So, if we used the incomplete and inconsistent dataset that we currently have and we based our AI algorithms on that then we’re going to be in significant danger of proliferating health inequalities. So, we need to be very careful with that around about our data that we have. We need to do a really good job of increasing and improving the quality and quantity of data that we have.

P4

Additionally, it was identified that, in part, due to the AI model not having enough data to learn from, this can lead to AI hallucination, which is where the AI tool produces a response that is false or misleading but is presented as fact:

[AI] does lead to false positives. [AI tools] are equally fraught with issues in the way that they work, not least because of their inherent ability to hallucinate and effectively make up results.

P1

However, despite these concerns, it was highlighted that due to rising patient demand, primary care could not continue in the way it has done, and that AI provided an opportunity to do things differently:

AI gives us the opportunity to think differently. So, there is an opportunity to explore things and try things differently and if we do it right there’s great potential to have success. …I use an Albert Einstein quote an awful lot, “In the midst of crisis, lies great opportunity.”

P4

## 5 Discussion

This study explored stakeholder's perceptions of the use of AI in a Primary Care setting in the Northwest of England. All stakeholders in this study were positive and emphasised the need to introduce AI into clinical practice in primary care. Participants supported the use of AI in providing a ‘clinical nudge’ to assist clinicians in their clinical decision making but not replace them. Nudge interventions have grown in interest in healthcare, particularly from a patient's perspective in relation to medication adherence and promoting healthy behaviours in chronic disease (Sumner et al. 2023). Nudges potentially change people's behaviour without eliminating options (Rooze 2009; Möllenkamp et al. 2009). In contrast to previous studies, this study supports the use of a nudge for clinicians from both the patient and clinical perspective. Previous studies highlight that healthcare providers are cautiously optimistic about using AI in administrative roles but hesitant about its clinical applications (Thornton et al. 2024). Fazakarley et al. (2023) reported that NHS clinicians are generally more comfortable with AI scheduling and data management, where AI’s potential to reduce time‐intensive repetitive tasks is clear. Reticence is identified when AI involvement is considered for diagnosis (Thornton et al. 2024). Blease et al. (2019) found that general practitioners (GPs) valued AI's potential to support, rather than replace, human judgement, believing that AI could augment clinical workflows but should remain subservient to the clinician's expertise. Aldosari et al. (2025) found that healthcare providers worry that excessive reliance on AI in diagnostics could erode essential diagnostic skills over time. The risk of deskilling represents a major caution for healthcare providers; although AI can aid in diagnosis, it must not replace human expertise (Aldosari et al. 2025). Moreover, Morrison (2021), emphasises that healthcare providers value the humanistic aspects of care, such as empathy, active listening and patient rapport. By assisting the clinician not replacing, this study confirmed that the potential to strengthen the consultation experience could be enhanced by an AI nudge rather than diminished.

Participants in this study described the potential for AI to explore noisy data, normally inaccessible or difficult to locate in a time limited consultation, and then present risk factors of a serious disease back to the clinician. Participants felt AI could be a clinical enabler, and enhance the patient consultation and patient safety by AI managing vast amount of data, linking important information from electronic patient records, facilitating earlier diagnosis and improving patient outcomes. Importantly, it could help support the generalists and less experienced clinicians with complex clinical reasoning. Not only could AI interrogate a large quality of noisy data relating to the patient's medical history but it could also summaries current clinical guidelines. Clinical guidelines have increasingly become an important part of clinical practice and influence evidence based clinical decisions on a daily basis (Woolf et al. 1999; Timmermans 2005). The ability of AI to summarise relevant up‐to‐date guidelines during a time limited patient consultation and present them to the clinician would ultimately enhance patient outcomes.

Machine learning models are now widely used across society to manage large datasets and problem solve in a variety of disciplines, but the literature highlights that they may create uncertainty generating unreliable predictions (Alzraiee and Niswanger 2024). One participant in this study with extensive experience of using AI in clinical practice concurred, describing problems relating to AI hallucinations and quality of data. Participants questioned who would be responsible and liable if things went wrong such as a serious condition being missed. Morrison (2021) emphasise the importance of clear, enforceable policies to define accountability and data privacy standards, which are essential for safe AI use in clinical settings. Codari's study, in particular, found that NHS staff were strongly in favour of comprehensive regulations, believing that these would provide the necessary assurances to support AI adoption without compromising patient safety. This call for regulation aligns with findings from the Frontiers AI Perception Study (Castagno and Khalifa 2020), which documented that many healthcare providers felt a need for guidelines that clarify liability, data handling, and ethical responsibilities when using AI in healthcare. Additional concerns were raised around connectivity in the NHS, both within and between primary and secondary care, in the context of an ageing NHS infrastructure, and concerns relating to cybersecurity. The problems with outdated information technology were highlighted in an independent investigation of the NHS in England, which attributed lack of capital investment and years of austerity to the NHS technological demise (Darzi 2024; Alderwick and Dunn 2024). Finally, the cost of AI and who pays, tech literacy, and impact on health inequalities were raised as further considerations.

The study's findings resonate with previous research, which collectively suggests that the use of AI in healthcare has potential, but a balanced approach is necessary and gaining an understanding of public and staff attitudes towards the use of AI in health care is important (Thornton et al. 2024). Comprehensive training and robust regulatory frameworks to ensure AI complements and assists rather than displaces human expertise are key. This multifaceted approach can help mitigate concerns about deskilling, support patient‐centred care, and establish a trustworthy foundation for AI integration in UK primary care settings. These findings lay the groundwork for future research into the development of AI in healthcare in a primary and community setting in the NHS. As Albert Einstein once said ‘In the midst of every crisis lies great opportunity, ’ participants in this study felt that the introduction of AI and machine learning in clinical settings promises improvements in the consultation experience of both the patient and the clinician. Importantly, patient safety, speed of accurate diagnosis and quality care are envisaged to make healthcare more responsive, efficient, effective and accessible for all.

## 6 Conclusion

This study provides unique insights from a 360° stakeholder's perspective into perceptions and acceptability of the use of AI tools in a Primary and Community Care setting in the Northwest of England. The concept of AI as a ‘clinical nudge’ to enable clinicians in decision making during time pressured consultations when dealing with vast amounts of noisy data in complex patient presentations is exciting and worthy of development. However, it must not replace the clinician but assist in decision making and for success to be realised the governance surrounding the use of AI must be robust and NHS aged infrastructure needs to be upgraded to be fit for purpose.

## Author Contributions

Conception or design of the work, data collection, data analysis and interpretation, drafting the article, critical revision of the article and final approval of the version to be published.

## Ethics Statement

The authors have nothing to report.

## Conflicts of Interest

The authors declare no conflicts of interest.

## Supporting information

Supporting Information S1

## Data Availability

The data that support the findings of this study are available on request from the corresponding author. The data are not publicly available due to privacy or ethical restrictions.
