# Supplementary material for: ‘In the Midst of Every Crisis, Lies Great Opportunity’: Perceptions of the Future Use of Artificial Intelligence in the UK NHS Primary Care
Source: Musculoskeletal Care. 2025 Apr 16;23(2):e70092. doi: 10.1002/msc.70092 (PMC12002359; doi:10.1002/msc.70092)
Supplement: Supplementary file 1 — Supporting Information S1 [file MSC-23-e70092-s001.docx]

**Topic guide**

1. Exploration of working (clinically) with AI in a health care setting

2. Explore benefits and facilitators (drawbacks/barriers) to using AI in a Primary Care setting

- Personally
- For wider health care

3. Explore thoughts about developing an AI system that would highlight to the clinician that a potential serious pathology may be the cause of the patients' symptoms.

- - Is it needed?
  - Would clinicians use it/want to use it?
  - Will it make a difference?
  - Is it feasible?

4. Explore perception of whether clinical/administrative/technical colleagues would support/embrace/use this technical development to enhance patient safety in clinical settings

- - Is it acceptable?
